# The lived experiences of primary caregivers supporting adolescents with non-suicidal self-injury: a qualitative systematic review and meta-aggregation

**DOI:** 10.3389/fpsyt.2026.1817553

**Published:** 2026-09-30

**Authors:** Xubin He, Xinghong Xu, Ping Yang, Chunyan Xie, Bo Yang

**Affiliations:** Chongqing Mental Health Center, Chongqing, China

**Keywords:** adolescents, lived experiences, non-suicidal self-injury, primary caregivers, meta-aggregation

## Abstract

**Objectives:**

To systematically identify, appraise, and synthesize qualitative evidence on the lived experiences, caregiving challenges, and support needs of primary caregivers of adolescents engaging in non-suicidal self-injury (NSSI).

**Methods:**

We searched PubMed, Embase, CINAHL, PsycINFO, Web of Science, CNKI, Wanfang Data, and CBM for English- and Chinese-language studies published through December 31, 2025. Two reviewers independently screened studies, appraised methodological quality using the JBI Critical Appraisal Checklist for Qualitative Research, and extracted findings with supporting illustrations. Evidence was synthesized using JBI meta-aggregation, and confidence in the synthesized findings was assessed using ConQual.

**Results:**

17 studies conducted across 5 countries and involving 395 primary caregivers were included. 77 findings were aggregated into 11 categories and 5 synthesized findings (1): Emotional shock, persistent distress, and stigma following NSSI discovery (2); Disruption of family relationships, functioning, and everyday life (3); Difficulties understanding NSSI and developing caregiving competence (4); Barriers to support and unmet needs for coordinated services; and (5) Adaptation, reflective change, and the emergence of resilience.

**Conclusion:**

The evidence portrays caregiving for adolescents with NSSI as a context-dependent and evolving process in which substantial emotional and practical burdens may coexist with gradual adaptation. These findings support evaluating non-blaming, family-centered care that strengthens caregiver well-being and competence and improves cross-sector coordination.

**Systematic review registration:**

https://www.crd.york.ac.uk/PROSPERO/, identifier CRD420251113508.

## 1 Introduction

Non-suicidal self-injury (NSSI) refers to the intentional and direct damage of one’s own body tissue without suicidal intent and for purposes not sanctioned by social or cultural norms; common methods include cutting, scratching, burning, biting, and hitting or banging oneself (1). The central distinction between NSSI and suicidal behavior is intent: NSSI occurs without an intention to die, whereas suicidal behavior involves at least some intent to end one’s life (2).

NSSI commonly emerges in early adolescence and becomes particularly prevalent during mid-adolescence (3). A meta-analysis involving 266,491 adolescents from 17 countries produced a pooled prevalence estimate of approximately 17.7% (4). Beyond direct tissue damage, NSSI is associated with substantial emotional distress, impaired academic and interpersonal functioning, poorer quality of life, and clinical difficulties, including depressive symptoms, emotion dysregulation, and substance use (5–7). Although NSSI is nonsuicidal by definition, it remains closely associated with suicidal ideation and an elevated risk of subsequent suicide attempts, making it an important clinical and public health concern in adolescent mental health (4, 8).

Because adolescents remain substantially dependent on their families for daily care, emotional support, and healthcare decisions, the consequences of NSSI rarely affect the young person alone. The family is both the setting in which NSSI is discovered, interpreted, and responded to and the environment in which professional help-seeking, treatment coordination, risk monitoring, and continuing support occur (9–12). A systematic review found relatively consistent associations between youth NSSI and low parental support, high psychological control, and reactive or punitive parenting (10). However, much of this evidence derives from observational studies and young people’s reports of parenting. The relationship between family processes and NSSI may therefore be bidirectional, and caregivers should not be positioned as a simple or unidirectional cause of the behavior. Once NSSI is discovered, the initial responses of parents and other primary caregivers may also influence whether adolescents disclose the behavior, when they seek formal help, and whether they remain willing to receive support (11). Nevertheless, many caregivers lack accurate knowledge of NSSI, effective communication strategies, and confidence in responding to risk, leaving them uncertain about how to protect the adolescent, preserve the relationship, and obtain appropriate professional help (11–13).

Providing such support can also have substantial consequences for caregivers themselves. In a comparative study, mothers of female adolescents who engaged in NSSI reported higher levels of depressive, anxiety, and stress symptoms than mothers in a nonclinical control group, as well as lower parental satisfaction than mothers in both clinical and nonclinical control groups (14). Other studies indicate that caregivers may experience shock, fear, self-blame, internalized stigma, and persistent anxiety. The need to monitor the possibility of further self-injury may also lead them to reorganize family routines, restrict social activities, or alter their employment, while financial strain, changes in family relationships, and inadequate support from mental health services may compound the burden of care (9, 11, 12, 15). Caregivers’ experiences should therefore not be regarded as a secondary consequence of adolescent NSSI. Rather, they constitute an important dimension of its family impact and of the caregiving process itself. Systematically integrating evidence on how caregivers understand NSSI, assume caregiving responsibilities, manage emotional disruption, and seek support is necessary for a fuller account of the burden associated with adolescent NSSI.

A growing body of qualitative research has examined the experiences of parents and other primary caregivers supporting adolescents who engage in NSSI. These studies have documented caregivers’ emotional responses to discovering the behavior, changes in caregiving roles and family relationships, attempts to manage risk, and difficulties encountered when interacting with mental health services (9, 11–13). However, the evidence remains dispersed across countries, cultures, and service settings, and studies differ in how they define self-injury, adolescence, and the caregiving role. Although individual qualitative studies provide detailed accounts of particular contexts, they cannot establish which experiences recur across caregiving settings or which are shaped by cultural expectations, family structures, and the availability of professional support. A systematic appraisal and synthesis of this evidence is therefore warranted.

Previous reviews have provided an important foundation for understanding how families respond to self-harm among young people, but their scope differs from that of the present review. Early reviews by Arbuthnott and Lewis and by Curtis et al. broadly examined parental factors, help-seeking, family impact, and the perspectives of both young people and family members; these reviews primarily used narrative approaches and extended beyond NSSI to broader forms of deliberate self-harm (16, 17). Mughal et al. subsequently conducted a systematic thematic synthesis of the experiences and needs of parents, relatives, and other supporters of young people aged 12–25 years who had self-harmed. Simes et al. synthesized the perspectives of both young people and caregivers affected by suicidality or self-harm, whereas Zhao et al. used meta-aggregation to examine parents’ experiences of caring for young people who self-harmed (18–20). Although these reviews made substantial contributions, their eligibility criteria were generally based on broad concepts of self-harm or suicide-related behavior. Some combined self-injury, self-poisoning, suicide attempts, and behaviors of uncertain intent, while others included wider age ranges and different types of supporting individuals. Their findings may therefore not fully capture the experiences that are specific to primary caregivers supporting adolescents whose behavior is explicitly identified as NSSI.

At the time of the present search, no completed qualitative evidence synthesis had been identified that focused exclusively on primary caregivers supporting adolescents who engage in explicitly defined NSSI. The present review therefore aimed to systematically identify, critically appraise, and synthesize qualitative evidence using meta-aggregation to examine these caregivers’ lived experiences. Particular attention was given to their emotional responses, changes in family and everyday life, caregiving challenges and coping processes, interactions with formal and informal sources of support, and unmet support needs. By integrating findings across cultural and caregiving contexts, the review sought to identify both recurring patterns and context-dependent differences, thereby providing a more coherent evidence base for future research, caregiver support, and the improvement of mental health services.

## 2 Methods

The review was conducted using JBI guidance for qualitative evidence synthesis and meta-aggregation, and its reporting followed the PRISMA 2020 statement (21, 22). The protocol was registered in PROSPERO (CRD420251113508).

### 2.1 Search strategy

The search was developed in three stages. First, a limited search in PubMed and CINAHL was used to examine the wording and indexing of relevant records. The resulting terms represented four concepts: primary caregivers or family members; adolescents or young people; NSSI and related self-harm terminology; and qualitative methods or lived experience. The strategy was then tailored for PubMed, Embase, CINAHL, PsycINFO, Web of Science, CNKI, Wanfang Data, and the Chinese Biomedical Literature Database (CBM). In the final stage, the reference lists of included studies were searched by hand. Broad expressions such as self-harm, deliberate self-harm, and self-injury were retained to reduce the risk of missing NSSI-focused research, but records identified through these expressions remained subject to the NSSI-specific eligibility rules described below. Searches covered database inception to December 31, 2025, and were limited to reports in English or Chinese. The complete PubMed strategy is reproduced in **Appendix I** in **Supplementary Material**.

### 2.2 Inclusion criteria

#### 2.2.1 Participants

Eligible studies reported qualitative data from primary caregivers—including parents, guardians, or close family members—who provided direct emotional, psychological, or practical support to a young person engaging in NSSI. Young people were operationalized as 10–24 years of age to reflect the extended developmental period described by Sawyer et al. (23).

#### 2.2.2 Phenomena of interest

Eligible findings addressed how primary caregivers understood, experienced, and managed support for a young person engaging in NSSI. This included caregivers’ emotional and psychological responses, changes in relationships, family roles, and everyday routines, caregiving knowledge and confidence, coping and help-seeking, encounters with services, and unmet support needs. For eligibility, NSSI was defined as intentional and direct damage to one’s own body tissue undertaken without an intention to die and for reasons not accepted as social or cultural practices. Fulfilment of the proposed DSM-5 diagnostic criteria for NSSI was not required (24).

Nonsuicidal intent was established from the definition and analytic framing used in each primary report; it was not assumed from the method of injury, its clinical severity, or the lack of a recorded suicide attempt. Reports using umbrella terms such as ‘self-harm’ were examined at full text and were retained only when the authors explicitly characterized the behavior as nonsuicidal or when NSSI-specific caregiver findings could be distinguished from findings about suicidal or intent-uncertain behavior. Studies combining these forms of self-injury without separable caregiver data were excluded.

#### 2.2.3 Context

No restriction was imposed on country, ethnicity, cultural background, or care setting. Eligible contexts included psychiatric inpatient and outpatient services, schools, community-based services, and caregiving in the home.

#### 2.2.4 Types of studies

Primary qualitative studies were included if they used a recognized qualitative methodology or approach, including phenomenology, grounded theory, ethnography, action research, thematic analysis, or qualitative description, and reported analyzable accounts obtained through methods such as interviews, diaries, field notes, or participant narratives. A qualitative component embedded in a mixed-methods study was eligible when its data and findings were reported separately and all other criteria were met. Quantitative-only studies, evidence syntheses, editorials, commentaries, letters, protocols, conference abstracts, and reports without retrievable full text or analyzable primary qualitative data were excluded. Gray literature remained eligible when it described a transparent qualitative design and contained analyzable primary data.

### 2.3 Study selection

Records were imported into EndNote 20 and duplicates were removed through automated matching followed by manual checking. Before formal screening, XBH and XHX piloted the eligibility criteria on 20 titles and abstracts; screening proceeded once agreement was at least 80% (25). The same reviewers then independently screened all titles and abstracts and evaluated potentially eligible full texts in JBI SUMARI (26). English- and Chinese-language reports were assessed in their original language. Exclusion reasons were documented, and disagreements were settled through discussion, with CYX or BY adjudicating any unresolved decision. Before the evidence set was finalized, every report retrieved through broad self-harm terminology was checked again against the operational definition of NSSI.

### 2.4 Assessment of methodological quality

XBH and XHX independently evaluated all included studies with the 10-item JBI Critical Appraisal Checklist for Qualitative Research (21). They first assessed a calibration set together to establish a shared interpretation of the checklist, after which each item was recorded as Yes, No, Unclear, or Not applicable. Differences were reconciled through discussion; CYX or BY was consulted when consensus could not be reached. Appraisal findings did not function as an inclusion threshold. Instead, identified limitations were carried forward when interpreting the synthesis and assigning ConQual confidence. Study-level appraisal decisions are shown in **Appendix II** in **Supplementary Material**.

### 2.5 Data extraction

XBH and XHX independently completed a standardized extraction form based on the JBI framework (21). PY subsequently checked the completed records for consistency and missing information. The form captured publication year and country; sample size; caregiver relationship and characteristics; the young people’s age range and relevant clinical background; study setting, methodology, and data-collection method; the phenomenon examined; the authors’ qualitative findings; and the accompanying illustrations, such as participant quotations. Extraction differences were resolved by discussion.

Before aggregation, each extracted finding was assigned a credibility level of Unequivocal, Credible, or Not Supported by judging how closely the authors’ finding corresponded to its supporting illustration (21). XBH and XHX compared their judgments and reached agreement through discussion, with PY resolving any outstanding differences. Consensus classifications were entered in JBI SUMARI (26).

### 2.6 Data synthesis

JBI meta-aggregation was applied to findings classified as Unequivocal or Credible (27). The reviewers compared the meaning of individual findings, assembled conceptually similar findings into categories, and then combined related categories into synthesized statements. Throughout this process, the synthesis was kept close to the interpretations presented by the primary-study authors rather than being used to generate a new theoretical account. Findings classified as Not Supported were to be withheld from aggregation and documented separately; no such finding remained in the final evidence set.

### 2.7 Assessing confidence in the findings

The JBI ConQual approach was used to judge confidence in each synthesized finding (28). All synthesized findings began at high confidence and could be downgraded for concerns about dependability, informed by the critical appraisal, or credibility, informed by the strength of the link between findings and their illustrations. XBH and XHX completed the assessments independently, PY verified them, and the reviewers resolved differences by consensus. Final confidence was reported as high, moderate, low, or very low. **Appendix V** in **Supplementary Material** contains the Summary of Findings table and the reasons for each rating.

## 3 Results

### 3.1 Study inclusion

Database searches identified 10,009 records. After 8,952 duplicates were removed, 1,057 records were screened, and 991 were excluded. 66 database reports were sought for retrieval, of which 17 could not be obtained. 49 database reports and 11 reports identified through citation and supplementary searching were assessed at full text. 43 reports were excluded for other eligibility reasons, resulting in 17 included studies (9, 11, 12, 29–42). The selection process is shown in **Figure 1**.

**Figure 1:**
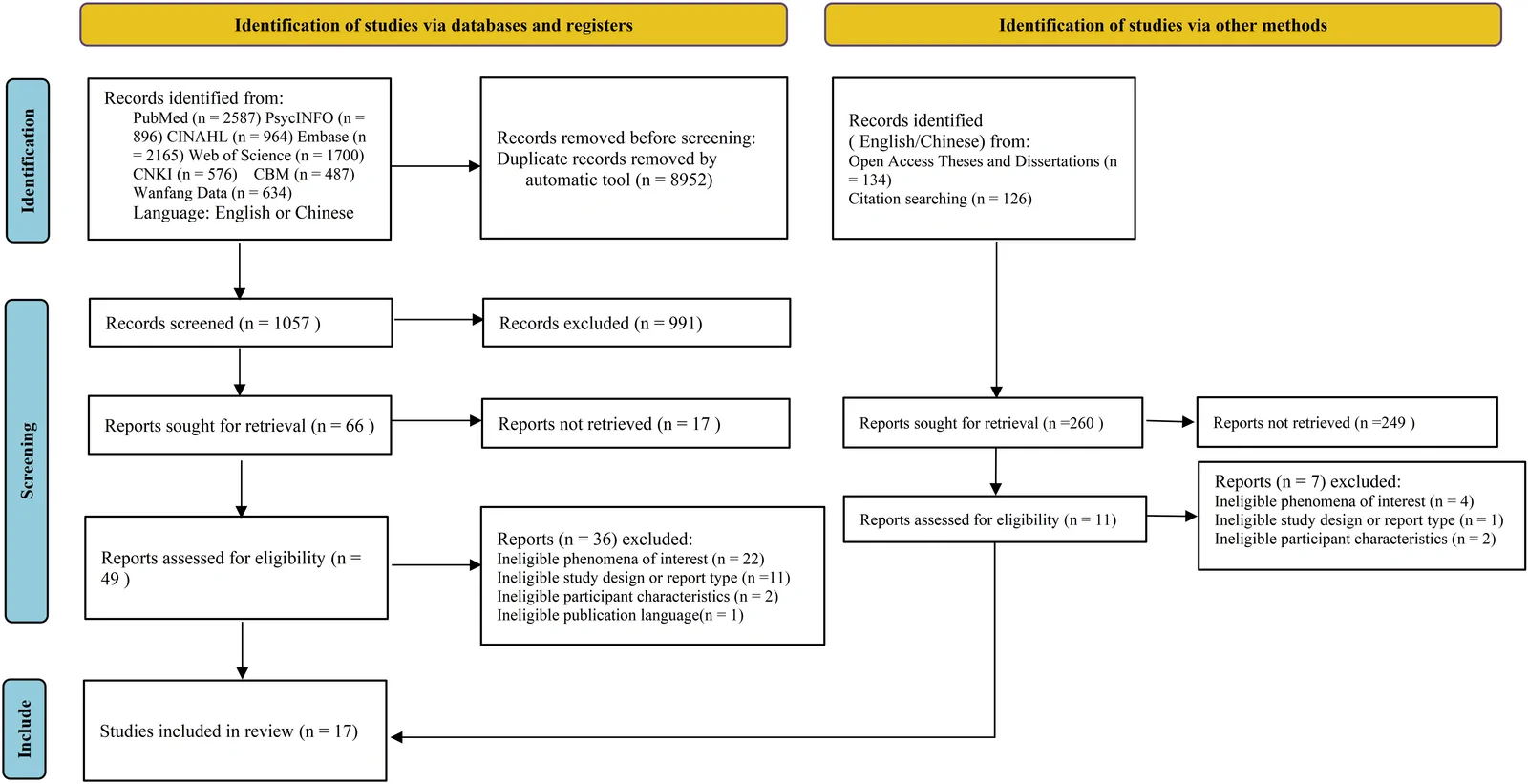
Preferred reporting items for systematic reviews and meta-analyses flow diagram.

### 3.2 Methodological quality

All 17 included studies were appraised with the JBI checklist (**Appendix II** in **Supplementary Material**). Individual studies received 7 to 10 “Yes” ratings across the 10 items. All studies demonstrated congruity between the research question, methodology, data collection, analysis, and interpretation. Five studies (29%) located the researcher culturally or theoretically, and five (29%) addressed the influence of the researcher on the research. Sixteen studies (94%) reported ethical approval; ethical status was unclear in one study.

### 3.3 Characteristics of included studies

The final evidence base comprised 16 qualitative studies and one mixed-methods study with a separately reported qualitative component (**Appendix III** in **Supplementary Material**). Thirteen studies were conducted in China; one multi-country study included participants from Australia and the United States; one additional study was conducted in the United States, one in Finland, and one in Thailand; the evidence therefore represented five countries. Publication years ranged from 2003 to 2025. Thirteen studies used phenomenological approaches (9, 11, 12, 33–42), one used thematic analysis (31), two used descriptive qualitative approaches (29, 30), and one used a mixed-methods design (32). Semi-structured or in-depth interviews were the most common data-collection methods.

The 17 studies included 395 primary caregivers, with study samples ranging from 3 to 135 participants. The caregiver sample was not restricted to parents. Relationship information was reported for 245 participants: 243 were parents and two were grandparents. Two studies, contributing 150 participants, used the broader term caregivers without providing a relationship-level breakdown. Accordingly, the term primary caregivers is used inclusively throughout this review.

### 3.4 Review findings

Seventy-seven findings were aggregated into 11 categories and 5 synthesized findings: (1) Emotional shock, persistent distress, and stigma following NSSI discovery; (2) Disruption of family relationships, functioning, and everyday life; (3) Difficulties understanding NSSI and developing caregiving competence; (4) Barriers to support and unmet needs for coordinated services; and (5) Adaptation, reflective change, and the emergence of resilience. Seventy-five findings (97.4%) were rated Unequivocal and two (2.6%) were rated Credible; none were rated Not Supported (**Appendix IV** in **Supplementary Material**). The two credible findings concerned teacher-student conflict and peer problems: their illustrations were relevant but lacked sufficient contextual detail for an unequivocal rating. All final classifications were reached by reviewer consensus. The synthesized findings describe recurring patterns in the included studies and should not be interpreted as universal experiences, prevalence estimates, or causal relationships. To strengthen the audit trail while limiting repetition, one concise supporting illustration is presented for each category. Additional verified quotations and the complete source-verification audit are provided in **Appendix IV** in **Supplementary Material**. Each quotation is followed by the participant identifier reported in the primary study and the corresponding reference number.

#### 3.4.1 Synthesized finding 1: emotional shock, persistent distress, and stigma following NSSI discovery

##### 3.4.1.1 Category 1: initial shock, self-blame, and crisis-related emotional responses

Caregivers often described discovering NSSI as unexpected and destabilizing, with immediate reactions of shock, fear, anger, helplessness, and self-blame (9, 11, 29, 31, 33, 36, 40, 41). Limited prior awareness of NSSI and uncertainty about how to respond were prominent features of these initial accounts.

“Well I had never even heard of it … so it just … scared the daylight out of me…” (P2, mother; 31)

##### 3.4.1.2 Category 2: persistent distress, fear, and emotional exhaustion

Distress often continued beyond the initial discovery. Caregivers described persistent fear of recurrence, heightened vigilance, guilt, sleep disturbance, and emotional exhaustion (9, 11, 12, 29, 34, 35, 41). Some also feared accidental death or possible future suicidal behavior; these fears were described alongside intensive monitoring and disruption of their own routines.

“…I didn’t sleep much the previous few days. I was afraid she would hurt herself.” (p20; 11)

##### 3.4.1.3 Category 3: affiliate stigma, concealment, and parental identity threat

Caregivers described feeling blamed, judged, or perceived as inadequate parents. Anticipated misunderstanding led some to conceal the adolescent’s NSSI, limit social contact, or withdraw from previously valued relationships (9, 12, 41, 42). Affiliate stigma was expressed through concealment, social withdrawal, and threats to parental identity.

“I did not tell any of my relatives, fearing that they would not understand…” (P3; 9)

#### 3.4.2 Synthesized finding 2: disruption of family relationships, functioning, and everyday life

##### 3.4.2.1 Category 4: communication barriers and changes in caregiver-adolescent interaction

Caregivers described difficulty communicating with the adolescent while attempting to balance protection, boundary-setting, and autonomy. Their accounts encompassed conflict or unmet emotional needs before NSSI was identified, as well as post-discovery increases in caution, monitoring, or accommodation, often linked to fear of recurrence (29, 31, 33, 40, 42).

“You just had to be so careful … It was like walking on eggshells with her.” (P17, mother; 31)

##### 3.4.2.2 Category 5: family relationship disruption and role strain

Caregivers reported changes across the wider family, including strained relationships, altered household routines, redistribution of responsibilities for treatment and supervision, and reduced attention to siblings or other family members (9, 11, 12, 29, 31, 32, 42). Reported role strain extended beyond the caregiver-adolescent dyad to couple relationships, sibling needs, and household responsibilities.

“…She says that we are too good to her sister and that everything is about her.” (P10; 9)

##### 3.4.2.3 Category 6: caregiving burden and restriction of everyday and social life

Caregivers described restrictions on employment, leisure, social participation, and self-care arising from supervision and treatment responsibilities, together with substantial financial pressure (9, 11, 12, 34, 37, 39). Physical exhaustion and reduced appetite were also reported, indicating that caregiving burden extended across occupational, social, physical, and financial domains.

“I quit my job … Now I mainly focus on treating my child’s disease…” (P8; 9)

#### 3.4.3 Synthesized finding 3: difficulties understanding NSSI and developing caregiving competence

##### 3.4.3.1 Category 7: meaning-making, attribution, and uncertainty about NSSI

Caregivers sought explanations for NSSI and attributed it to emotion regulation, communication of distress, interpersonal conflict, developmental change, or broader mental-health difficulties (12, 30, 32, 33, 40). Uncertainty often persisted regarding the adolescent’s intent, recovery, and future needs. Some attempted to distinguish NSSI from suicidal behavior by reference to injury severity, reflecting uncertainty in their understanding rather than a clinically reliable assessment of intent.

“Her self-injury is, I think, a kind of resistance to mental pain … I knew it was not suicide…” (P04; 33)

##### 3.4.3.2 Category 8: knowledge gaps and caregiving competence deficits

Beyond uncertainty about the meaning of NSSI, caregivers described feeling unprepared to act. They lacked practical guidance on managing safety, communicating with the adolescent, supporting treatment, and providing care at home (9, 12, 31, 34, 36, 37, 39). Inadequate information during treatment and discharge was accompanied by low confidence in postdischarge caregiving.

“I had no idea that she did that in response to being in pain … I thought it was just a one time … I didn’t know that it was self-injury … I had no idea.” (P1, mother; 31)

#### 3.4.4 Synthesized finding 4: barriers to support and unmet needs for coordinated services

##### 3.4.4.1 Category 9: barriers in professional, school, and community support

Caregivers described barriers across health, school, and community settings, including limited local mental-health resources, fragmented referral pathways, dismissive or stigmatizing interactions, and inconsistent responses (9, 12, 29, 31, 41, 42). Some felt excluded from treatment communication or left to identify appropriate expertise without professional guidance.

“I pretty much right away tried to get her into a counsellor but … I was completely on my own…” (P7, mother; 31)

##### 3.4.4.2 Category 10: needs for psychoeducation, practical guidance, and multidimensional support

Caregivers expressed needs for accessible information about NSSI, practical guidance on safety and communication, support for their own emotional well-being, and continuity after discharge (9, 31, 35–37, 39, 42). They also requested crisis contacts and clearer coordination among families, health services, schools, and community services.

“Is there a parent’s guide to what to do when your daughter cuts? … I wish I had seen it [laughs].” (P6, mother; 31)

#### 3.4.5 Synthesized finding 5: adaptation, reflective change, and the emergence of resilience

##### 3.4.5.1 Category 11: reflective growth, coping adjustment, and reconstruction of caregiving efficacy

Some caregivers described gradually revising expectations, modifying communication or parenting practices, seeking information, developing coping skills, and drawing on family or peer support (9, 11, 12, 31, 33–40). These changes coexisted with continuing distress and uncertainty, suggesting adaptation without implying that caregiving difficulties had resolved.

“…I will change myself. This family can be more democratic, I will listen to her…” (P18; 33)

### 3.5 Confidence in the synthesized findings

All five synthesized findings were downgraded by one level for dependability because of methodological heterogeneity and limited reporting of researcher positioning and reflexivity. Synthesized Findings 1, 2, 3, and 5 were supported exclusively by unequivocal findings and received high credibility and an overall ConQual rating of moderate. Synthesized Finding 4 included two credible findings related to school and peer problems; its credibility was rated moderate, and its overall ConQual rating was low (**Appendix V** in **Supplementary Material**).

## 4 Discussion

This review synthesized 17 NSSI-specific studies involving 395 primary caregivers and identified five interrelated domains of experience: emotional shock, persistent distress, and stigma following the discovery of NSSI; disruption to family relationships and everyday life; difficulties understanding NSSI and developing caregiving competence; barriers to obtaining coordinated support; and, for some caregivers, adaptation and emerging resilience. Whereas previous reviews primarily examined broader forms of self-harm encompassing different types of intent, the present review applied an explicit NSSI-specific eligibility boundary and focused on primary caregivers supporting adolescents. It therefore provides a more differentiated account of the emotional, relational, practical, and service-related demands of caregiving in the context of explicitly identified NSSI.

### 4.1 Emotional shock, persistent distress, and affiliate stigma

Caregivers’ emotional distress extended beyond initial shock and self-blame to persistent vigilance, sleep disturbance, guilt, and physical and emotional exhaustion. This finding is consistent with previous evidence showing clinically significant anxiety and depressive symptoms among some parents of adolescents with NSSI (43, 44). This evidence supports incorporating an assessment of caregiver well-being into family-centered NSSI care. However, existing research does not establish how caregiver distress affects caregiving behaviors or adolescent outcomes.

Affiliate stigma added to caregivers’ social and identity-related burden. Caregivers described anticipating blame, concealing NSSI, and withdrawing from social contact; previous qualitative studies have similarly reported experiences of secrecy and shame (13). These findings support further evaluation of nonjudgmental NSSI education, safety guidance, and emotional support for caregivers.

### 4.2 Family relationships, role strain, and everyday disruption

The second synthesized finding showed that NSSI became embedded in family life through changes in communication, monitoring, household roles, employment, social activities, and attention to other family members. Some caregivers became highly cautious or accommodating because they feared that disagreement or limit-setting might precipitate another episode of NSSI. Others increased supervision or restricted access to potentially harmful objects. These responses may be understood as caregivers’ attempts to manage risk under conditions of uncertainty, but they may also create tensions concerning privacy, autonomy, and trust.

The temporal relationship between family processes and NSSI is complex. Evidence suggests that harsh punishment, poor attachment, family dysfunction, parenting stress, or parental hostility may precede the subsequent onset of NSSI among adolescents. In addition, adolescent NSSI may predict later changes in perceived parental rule-setting, control, or monitoring (10, 45–50). However, reciprocal effects have not been consistently demonstrated across studies. The caregiver accounts synthesized in this review should therefore not be interpreted as indicating either that family difficulties necessarily caused NSSI or that all changes in family relationships resulted from NSSI.

Future family-focused support should address communication, safety, autonomy, and role strain while considering the effects of caregiving on siblings, couple relationships, employment, and everyday routines. Such support requires prospective evaluation and should not presume a single family deficit or causal pathway.

### 4.3 Understanding NSSI and developing caregiving competence

Caregiver uncertainty arose from both limited knowledge and multiple practical caregiving demands, including understanding NSSI and potential suicide risk, communicating with the adolescent, maintaining safety at home, supporting treatment, and knowing when and where to seek help. Accordingly, factual information alone may be insufficient to strengthen caregivers’ confidence in providing care.

Previous research suggests that NSSI may function to regulate distress or communicate needs that are difficult to express directly (51). Reviews of the broader self-harm literature indicate that caregivers’ emotions and knowledge can shape how they provide support, while a qualitative evaluation of online ERITA found that caregivers valued a dedicated caregiver course and therapist contact (18, 52, 53). These studies support co-designing future interventions with adolescents, caregivers, and professionals and including practical safety guidance, discharge preparation, and clear routes to follow-up professional advice. However, the effectiveness of specific components should be established in prospective evaluations.

### 4.4 Service barriers and unmet needs for coordinated support

Caregivers reported limited local resources, fragmented referral pathways, dismissive interactions with professionals, and inconsistent responses from schools or communities. Previous studies have similarly found that caregivers experienced difficulties obtaining relevant explanations, communicating with professionals, and accessing support for their own well-being (18, 53). Taken together, these findings suggest that families may be expected to assume substantial responsibility for monitoring and crisis management without necessarily receiving adequate preparation or continuity of support. Future work should therefore develop and test clearer referral pathways and discharge-planning procedures while clarifying the responsibilities of healthcare institutions, schools, and community services.

### 4.5 Conditional adaptation and emerging resilience

Despite continuing distress and uncertainty, some caregivers reported gradually revising their expectations, changing how they communicated, actively seeking relevant information, and becoming more confident in providing care (9, 11, 12, 31, 33–40). These accounts are more consistent with conditional adaptation or benefit finding than with complete recovery from caregiving burden. They do not imply that NSSI was beneficial, that all caregivers should be expected to adapt, or that changes within the family caused adolescent recovery. Future research should evaluate strengths-informed approaches that recognize caregivers’ existing knowledge and coping capabilities while continuing to assess their emotional distress and unmet needs.

## 5 Limitations

This review has several limitations. First, only English- and Chinese-language studies were included. Second, 13 of the 17 studies were conducted in China, limiting transferability to other cultural and service contexts. Third, the operational requirement for clearly identifiable nonsuicidal intent may have excluded clinically relevant studies using broader self-harm terminology or reporting mixed or fluctuating intent; however, including inseparable mixed samples would have undermined the NSSI-specific purpose of the review. Fourth, the 10–24-year age range extends beyond conventional definitions of adolescence and should be considered when interpreting applicability. Fifth, limited reporting of researcher positioning and reflexivity reduced dependability, and Synthesized Finding 4 had low overall ConQual confidence. Finally, initial reviewer agreement for credibility ratings could not be quantified because separate initial classifications were not retained, although all final decisions were reached by consensus.

## 6 Conclusions

The review identified five related domains of caregiver experience: emotional shock, persistent distress, and stigma; disruption to family relationships, functioning, and everyday life; difficulties understanding NSSI and developing caregiving competence; barriers to support and unmet needs for coordinated services; and adaptation, reflective change, and emerging resilience. The findings indicate that caregivers may need support for their own emotional well-being as well as practical guidance for communication, safety, and care coordination. Because the evidence is context-dependent and does not establish causality, services should use non-blaming, family-centered assessment and evaluate proposed supports in partnership with adolescents and caregivers.

## Data Availability

The original contributions presented in the study are included in the article/**Supplementary Material**. Further inquiries can be directed to the corresponding author.
