# Supplementary material for: The lived experiences of primary caregivers supporting adolescents with non-suicidal self-injury: a qualitative systematic review and meta-aggregation
Source: Front Psychiatry. 2026 Sep 30;17:1817553. doi: 10.3389/fpsyt.2026.1817553 (PMC13662121; doi:10.3389/fpsyt.2026.1817553)
Supplement: Supplementary file 1 [file Table1.docx]

**Appendix I: Search strategy**

| **PubMed** | | |
| --- | --- | --- |
| **Search conducted: December 31, 2025** | | |
| **Search** | **Search query** | **Records retrieved** |
| **#1** | parents[MeSH Terms] OR parent*[Title/Abstract] OR carer*[Title/Abstract] OR "Support Person"[Title/Abstract] OR adult*[Title/Abstract] OR relative*[Title/Abstract] OR Relation[Title/Abstract] OR Guardian[Title/Abstract] OR "family relation*"[Title/Abstract] OR caregiver*[Title/Abstract] OR "family member*"[Title/Abstract] OR famil*[Title/Abstract] OR mother*[Title/Abstract] OR father*[Title/Abstract] | 6,126,150 |
| **#2** | adolescent[MeSH Terms] OR "Young Person"[Title/Abstract] OR "Young People"[Title/Abstract] OR "Young Adult"[Title/Abstract] OR teen*[Title/Abstract] OR adolesc*[Title/Abstract] OR youth*[Title/Abstract] OR student*[Title/Abstract] OR "middle school student"[Title/Abstract] | 2,968,914 |
| **#3** | "self injurious behavior"[MeSH Terms] OR "self-harm"[Title/Abstract] OR "self harm*"[Title/Abstract] OR "self cut*"[Title/Abstract] OR "self-destruct"[Title/Abstract] OR "nonsuicidal self injur*"[Title/Abstract] OR "deliberate self harm"[Title/Abstract] OR "self mutil*"[Title/Abstract] OR "self inflicted injur*"[Title/Abstract] OR "self inflict*"[Title/Abstract] OR DSH[Title/Abstract] OR NSSI[Title/Abstract] OR "self injur*"[Title/Abstract] | 106,800 |
| **#4** | "focus groups"[MeSH Terms] OR "interviews as topic"[MeSH Terms] OR "narration"[MeSH Terms] OR qualitative research[MeSH Terms] OR "qualitative studies"[Title/Abstract] OR "phenomenological research"[Title/Abstract] OR "ethnographic research"[Title/Abstract] OR "grounded theory"[Title/Abstract] OR "mixed method* research"[Title/Abstract] OR "interview"[Title/Abstract] OR "semi-structured interview"[Title/Abstract] OR "focus groups"[Title/Abstract] OR "thematic analysis"[Title/Abstract] OR "content analysis"[Title/Abstract] OR "narratives"[Title/Abstract] OR "qualitative approach*" OR "descriptive study*" OR "experiences"[Title/Abstract] OR "perceptions"[Title/Abstract] OR "attitudes"[Title/Abstract] | 1,045,238 |
| **#5** | #1 AND #2 AND #3 AND #4 | 2,749 |
| #6 | #5 AND ("1900/01/01"[Date - Publication] : "2025/12/31"[Date - Publication]) | 2,616 |
| Limited to English- and Chinese-language studies published through December 31, 2025. | | |
