# Supplementary material for: The lived experiences of primary caregivers supporting adolescents with non-suicidal self-injury: a qualitative systematic review and meta-aggregation
Source: Front Psychiatry. 2026 Sep 30;17:1817553. doi: 10.3389/fpsyt.2026.1817553 (PMC13662121; doi:10.3389/fpsyt.2026.1817553)
Supplement: Supplementary file 2 [file Table2.docx]

**Appendix II: Critical appraisal of the included studies**

| **Study** | **Q1** | **Q2** | **Q3** | **Q4** | **Q5** | **Q6** | **Q7** | **Q8** | **Q9** | **Q10** |
| --- | --- | --- | --- | --- | --- | --- | --- | --- | --- | --- |
| Yip et al. (2003) (29) | Y | Y | Y | Y | Y | N | N | Y | U | Y |
| Rissanen et al. (2008) (30) | Y | Y | Y | Y | Y | Y | Y | Y | Y | Y |
| Kelada et al. (2016) (31) | Y | Y | Y | Y | Y | N | N | Y | Y | Y |
| Whitlock et al. (2018) (32) | Y | Y | Y | Y | Y | N | N | Y | Y | Y |
| Fu et al. (2020) (11) | Y | Y | Y | Y | Y | N | N | Y | Y | Y |
| Wang et al. (2022) (33) | Y | Y | Y | Y | Y | N | N | Y | Y | Y |
| Zhang et al. (2023) (34) | Y | Y | Y | Y | Y | N | N | Y | Y | Y |
| Xie et al. (2023) (35) | Y | Y | Y | Y | Y | N | N | Y | Y | Y |
| Gao et al. (2023) (36) | Y | Y | Y | Y | Y | Y | Y | Y | Y | Y |
| Wang et al. (2024) (37) | Y | Y | Y | Y | Y | Y | Y | Y | Y | Y |
| Li et al. (2024) (38) | Y | Y | Y | Y | Y | N | N | Y | Y | Y |
| Song et al. (2024) (39) | Y | Y | Y | Y | Y | Y | Y | Y | Y | Y |
| Gao et al. (2024) (40) | Y | Y | Y | Y | Y | N | N | Y | Y | Y |
| Wang et al. (2024) (12) | Y | Y | Y | Y | Y | Y | Y | Y | Y | Y |
| Li et al. (2025) (41) | Y | Y | Y | Y | Y | N | N | Y | Y | Y |
| Hu et al. (2025) (9) | Y | Y | Y | Y | Y | N | N | Y | Y | Y |
| Auernaruemonsuk et al. (2025) (42) | Y | Y | Y | Y | Y | N | N | Y | Y | Y |
| **Total (%)** | **100** | **100** | **100** | **100** | **100** | **29** | **29** | **100** | **94** | **100** |

Y, yes; N, no; U, unclear. JBI Critical Appraisal Checklist for Qualitative Research.

Q1 = Is there congruity between the stated philosophical perspective and the research methodology?
Q2 = Is there congruity between the research methodology and the research question or objectives?
Q3 = Is there congruity between the research methodology and the methods used to collect data?
Q4 = Is there congruity between the research methodology and the representation and analysis of data?
Q5 = Is there congruity between the research methodology and the interpretation of results?
Q6 = Is there a statement locating the researcher culturally or theoretically?
Q7 = Is the influence of the researcher on the research, and vice-versa, addressed?
Q8 = Are participants, and their voices, adequately represented?
Q9 = Is the research ethical according to current criteria or, for recent studies, is there evidence of ethical approval by an appropriate body?
Q10 = Do the conclusions drawn in the research report flow from the analysis, or interpretation, of the data?
