# Supplementary material for: The lived experiences of primary caregivers supporting adolescents with non-suicidal self-injury: a qualitative systematic review and meta-aggregation
Source: Front Psychiatry. 2026 Sep 30;17:1817553. doi: 10.3389/fpsyt.2026.1817553 (PMC13662121; doi:10.3389/fpsyt.2026.1817553)
Supplement: Supplementary file 3 [file Table3.docx]

**Appendix III: Characteristics of the included studies**

| **Author (year)** | **Country** | **Methodology** | **Participants and context** | **Sample size** | **Data collection** | **Phenomenon of interest** | **Summary of main findings** |
| --- | --- | --- | --- | --- | --- | --- | --- |
| Yip et al. (2003) (29) | China (Hong Kong SAR) | Descriptive qualitative study | Parents of students with NSSI behaviors from three secondary schools in Hong Kong. | 3 mothers | In-depth interviews | To explore parental influence on and reactions to adolescent NSSI. | Nine themes: Parental Influence on Self-Cutting: Unhappy Childhood and Family Life; Problems in Parent-Child Communication; Aversive Impact of an Unhappy Childhood and Parent-Child Communication; Parental Response to Adolescents' Self-Cutting: Discovery of Children's Self-Cutting; Feelings Toward Children's Self-Cutting; Communication With Their Children; Dealing With Parent-Child Conflicts Behind Self-Cutting; Dealing With Teacher-Student Conflicts Behind Self-Cutting; and Dealing With Their Children's Peer Problems and Their Relationship to Self-Cutting. |
| Rissanen et al. (2008) (30) | Finland | Descriptive qualitative study | Parents contacted through an existing study on adolescent NSSI. | 4 caregivers (1 father, 3 mothers) | Individual interviews | To describe parents' perceptions of adolescent NSSI. | Four themes: The phenomenon of NSSI; Factors contributing to NSSI; Purposes of NSSI; and Sequels of NSSI. |
| Kelada et al. (2016) (31) | Australia and United States | Thematic analysis | Parents recruited in two studies through high schools, flyers, or online advertisements. | 38 parents (Australia: 16; United States: 22) | Semi-structured interviews | To assess parental reactions to NSSI discovery and its impact on health, parenting, and interactions with professionals. | Study 1: six themes—Initial Responses to NSSI; Parents' Experience; Professional Help for Their Adolescent; Negative Interactions With Professionals; Parents' Perceived Helpfulness; and Least Helpful Parental Responses. Study 2: three themes—Lack of Knowledge About NSSI; Uncertainty About How to Interact With Their Child After NSSI; and Negative Experiences With Mental Health Professionals. |
| Whitlock et al. (2018) (32) | United States | Mixed-methods study | Parents were recruited through the Self-Injury and Recovery Resources listserv and web-based parent networks. | 135 primary caregivers; relationship breakdown not reported | Web-based survey; qualitative data were obtained through open-ended items and analyzed using the constant comparative method. | To examine parents’ perceptions of the positive and negative effects of their child’s NSSI on themselves, their child, and the parent–child relationship. | Three themes: Impact on family relationships in general; Impact on parent self-perception, well-being, and functioning; and Parent perception of child well-being and recovery (or lack thereof). |
| Fu et al. (2020) (11) | China | Phenomenological study | Parents of adolescents with NSSI in a pediatric psychiatric ward of a tertiary hospital. | 20 caregivers (16 mothers, 4 fathers) | Semi-structured interviews | To explore parental attitudes toward adolescent NSSI and its impact on the family. | Three themes: Attitudes Toward Children's NSSI Behaviors; Coping Strategies of Parents; and Impacts on the Family. |
| Wang et al. (2022) (33) | China | Phenomenological study | Mental health center of a tertiary hospital in Chengdu, Sichuan. | 24 caregivers (18 mothers, 6 fathers) | Semi-structured interviews | To explore parents' lived experiences of recurrent adolescent NSSI. | Three themes: Attribution of NSSI; Perceptions of NSSI; and Coping Responses to NSSI. |
| Zhang et al. (2023) (34) | China | Phenomenological study | Pediatric ward of a tertiary Grade A psychiatric hospital in Wuhan. | 10 caregivers (7 mothers, 2 fathers, 1 grandmother) | In-depth interviews | To understand the caregiving experiences of families of adolescents with psychiatric disorders and NSSI. | Four themes: Insufficient Cognition of NSSI; High Family Pressure; Complex Negative Emotions; and Reflection and Growth. |
| Xie et al. (2023) (35) | China | Phenomenological study | Parents of adolescents with NSSI in a tertiary Grade A hospital in Guangzhou. | 19 caregivers (12 mothers, 7 fathers) | In-depth interviews | To analyze the caregiving and coping experiences of parents of adolescents with NSSI. | Four themes: Multiple Negative Emotional Experiences; Creating a Positive Family Environment Through Various Means; Lack of Effective Coping Strategies and Limited Problem-Solving; and Insufficient External Support and Desire for More Assistance. |
| Gao et al. (2023) (36) | China | Phenomenological study | Caregivers of adolescents with mood disorders and NSSI in a tertiary Grade A psychiatric hospital in Henan. | 11 caregivers (9 mothers, 2 fathers) | Semi-structured interviews | To explore changes in caregiving difficulties across stages of illness. | Five themes: Diagnosis stage—complex reactions and helplessness; Hospitalization stage—limited knowledge and caregiving difficulties; Discharge preparation—low confidence and lowered expectations; Adjustment stage—insufficient skills and desire for professional support; and Adaptation stage—negative coping and persistent difficulties. |
| Wang et al. (2024) (37) | China | Phenomenological study | Caregivers of adolescents with mood disorders and NSSI at Beijing Huilongguan Hospital. | 14 caregivers (11 mothers, 3 fathers) | In-depth interviews | To explore the experiences and feelings of caregivers of adolescents with mood disorders and NSSI. | Five themes: Multiple Negative Emotional Experiences; Heavy Caregiving Burden; Lack of Caregiving Capacity; Personal and Family Growth; and Longing for Professional Help. |
| Li et al. (2024) (38) | China | Phenomenological study | Caregivers of adolescents with NSSI in an open ward of a psychiatric hospital in Henan. | 15 primary caregivers; relationship breakdown not reported | In-depth interviews | To investigate benefit finding among family caregivers of adolescents with NSSI. | Four themes: Perceived Meaning of Life; Improvement of Capabilities; Cognitive Transformation; and Perceived Support From Others. |
| Song et al. (2024) (39) | China | Phenomenological study | Primary caregivers of adolescents with depression and NSSI in a tertiary Grade A hospital in Nanyang. | 10 caregivers (6 mothers, 3 fathers, 1 grandfather) | Semi-structured interviews | To explore the caregiving experiences of caregivers of adolescents with depression and NSSI. | Three themes: Heavy Caregiving Burden; Lack of Illness-Related Knowledge and Caregiving Capacity; and Need for Multifaceted Support. |
| Gao et al. (2024) (40) | China | Phenomenological study | Parents of adolescents with depression at Nantong Fourth People's Hospital. | 15 caregivers (10 mothers, 5 fathers) | Semi-structured interviews | To explore parents' psychological experiences of adolescent depression and NSSI. | Five themes: Previous Focus on Work and Neglect of Emotional Needs; Intense Verbal Reactions and Self-Blame Upon Discovery; Anxiety and Fear of Stigma During Early Treatment; Reluctantly Lowering Expectations and Attempting to Accommodate the Child; and Ambivalence and Concern About the Future During Recovery. |
| Wang et al. (2024) (12) | China | Phenomenological study | A tertiary psychiatric hospital in Beijing. | 21 caregivers (16 mothers, 5 fathers) | Semi-structured interviews | To explore parents' experiences during the COVID-19 pandemic. | Four themes: Negative Experiences; High Caregiving Burden; Lack of Caregiving Capacity; and Resilience. |
| Li et al. (2025) (41) | China | Phenomenological study | Pediatric psychiatric clinic of the Affiliated Hospital of Hangzhou Normal University. | 18 caregivers (10 mothers, 8 fathers) | In-depth interviews | To explore affiliate stigma among parents of adolescents with NSSI. | Three themes: Initial Cognitive Conflict; Manifestations of Affiliate Stigma; and Negative Coping With Affiliate Stigma. |
| Hu et al. (2025) (9) | China | Phenomenological study | Parents of adolescents with NSSI in a tertiary hospital in Shanxi. | 18 caregivers (13 mothers, 5 fathers) | Semi-structured interviews | To explore parents' psychological experiences from an ecological-systems perspective. | Four themes: Microsystem—coexisting caregiver distress and growth (persistent negative emotions, positive experiences after self-adjustment, learning about mental illness, and improved illness-management capabilities); Mesosystem—lifestyle changes (withdrawal from social life, effects on other children, financial burden, and changes in family atmosphere); Exosystem—weak support systems (limited psychiatric-service resources and a desire for more support); and Macrosystem—cultural environment. |
| Auernaruemonsuk et al. (2025) (42) | Thailand | Phenomenological study | Parents of adolescents and young adults receiving NSSI-related psychiatric care in Bangkok. | 20 parents (15 mothers, 5 fathers) | Semi-structured interviews | To explore family experiences of receiving psychiatric treatment for NSSI. | Five themes: NSSI as a trigger for changes in relationships between families and young people, often linked to emotional neglect and high expectations; Negative effects on families and strategies for adjustment and problem-solving; School responses to NSSI, ranging from support to stigmatization; Other perspectives on NSSI characterized by both encouragement and stigma; and Mental health professionals' assistance in providing psychoeducation and facilitating communication to help families manage NSSI. |

*Note. Reference numbers correspond to the manuscript. NSSI, non-suicidal self-injury.*
