# Supplementary material for: The lived experiences of primary caregivers supporting adolescents with non-suicidal self-injury: a qualitative systematic review and meta-aggregation
Source: Front Psychiatry. 2026 Sep 30;17:1817553. doi: 10.3389/fpsyt.2026.1817553 (PMC13662121; doi:10.3389/fpsyt.2026.1817553)
Supplement: Supplementary file 4 [file Table4.docx]

**Appendix IV: Synthesis of study findings, categories, and synthesized findings**

Credibility assessment audit. Seventy-five findings were classified as Unequivocal (U), two as Credible (C), and none as Not Supported (NS). The two Credible ratings applied to “Dealing With Teacher-Student Conflicts” and “Dealing With Peer Problems” because the supporting illustrations were directionally relevant but lacked sufficient contextual detail for an Unequivocal rating. Final classifications were reached by reviewer consensus.

| **Synthesized finding** | **Category** | **Study finding** |
| --- | --- | --- |
| Synthesized Finding 1: Emotional shock, persistent distress, and stigma following NSSI discovery | Category 1: Initial shock, self-blame, and crisis-related emotional responses | Discovering self-harm (U) |
|  |  | Parental Response to Discovery (U) |
|  |  | Initial Responses to NSSI (U) |
|  |  | Diagnosis phase: complex emotional reactions and helplessness (U) |
|  |  | Pre-treatment anxiety and reluctance to seek medical help (U) |
|  |  | Initial cognitive conflict (U) |
|  |  | Intense verbal reactions and self-blame upon discovery (U) |
|  |  | Attitudes to children's NSSI behaviors (U) |
|  | Category 2: Persistent distress, fear, and emotional exhaustion | Feelings Toward Children’s Self-Cutting (U) |
|  |  | Multiple negative emotional experiences (U) |
|  |  | Complex negative emotions (U) |
|  |  | Micro-system—coexistence of caregiver distress and growth (U) |
|  | Category 3: Affiliate stigma, concealment, and parental identity threat | Manifestations of affiliate stigma (U) |
|  |  | Negative coping with affiliate stigma (U) |
|  |  | Other perspectives characterized by stigma (U) |
| Synthesized Finding 2: Disruption of family relationships, functioning, and everyday life | Category 4: Communication barriers and changes in caregiver-adolescent interaction | Uncertain about interaction after NSSI (U) |
|  |  | Problems in Parent–Child Communication (U) |
|  |  | Aversive Impact of Communication (U) |
|  |  | Communication With Their Children (U) |
|  |  | Dealing With Parent–Child Conflicts (U) |
|  |  | Caregivers’ retrospective accounts of relationship changes associated with emotional neglect or high expectations (U) |
|  |  | Caregivers’ retrospective accounts of unmet emotional needs before NSSI was identified (U) |
|  | Category 5: Family relationship disruption and role strain | Impacts on family (U) |
|  |  | Impact on family relationships (U) |
|  |  | Negative impacts and adjustment strategies (U) |
|  |  | Caregivers’ retrospective accounts of unhappy family life (U) |
|  | Category 6: Caregiving burden and restriction of everyday and social life | Meso-system—lifestyle changes (social life, financial burden) (U) |
|  |  | Heavy caregiving burden (U) |
|  |  | Burden of care (U) |
|  |  | High caregiving burden (U) |
|  |  | High family pressure (U) |
| Synthesized Finding 3: Difficulties understanding NSSI and developing caregiving competence | Category 7: Meaning-making, attribution, and uncertainty about NSSI | Perceptions of NSSI (U) |
|  |  | Attribution of NSSI (U) |
|  |  | Phenomenon of self-mutilation (U) |
|  |  | Factors contributing (U) |
|  |  | Purposes (U) |
|  |  | Sequels (U) |
|  |  | Perception of child well-being and recovery (U) |
|  |  | Ambivalence and decision difficulties during recovery, worrying about the child's future growth (U) |
|  | Category 8: Knowledge gaps and caregiving competence deficits | Lack of knowledge about NSSI (U) |
|  |  | Hospitalization stage: limited illness and parenting knowledge and difficulty caring for the child (U) |
|  |  | Insufficient cognition of NSSI (U) |
|  |  | Lack of disease-related knowledge and caregiving capacity (U) |
|  |  | Lack of caregiving capacity (U) |
|  |  | Insufficient illness-related caregiving capacity (U) |
| Synthesized Finding 4: Barriers to support and unmet needs for coordinated, empowering services | Category 9: Barriers in professional, school, and community support | Exo-system—weak support system (U) |
|  |  | Macro-system—cultural environment (U) |
|  |  | Negative experiences with professionals (U) |
|  |  | Negative Interactions with Professionals (U) |
|  |  | Negative experience (U) |
|  |  | School responses: from support to stigmatization (U) |
|  |  | Dealing With Teacher–Student Conflicts (C) |
|  |  | Dealing With Peer Problems (C) |
|  | Category 10: Needs for psychoeducation, practical guidance, and multidimensional support | Mental health professionals’ assistance (U) |
|  |  | Adjustment stage: insufficient caregiving skills, longing for professional support (U) |
|  |  | Professional Help (U) |
|  |  | Longing for professional help (U) |
|  |  | Need for multi-faceted support (U) |
|  |  | Discharge preparation stage: lack of home-care confidence (U) |
|  |  | Insufficient external support, parents hoping for more help (U) |
| Synthesized Finding 5: Adaptation, reflective change, and the emergence of resilience | Category 11: Reflective growth, coping adjustment, and reconstruction of caregiving efficacy | Personal and family growth (U) |
|  |  | Perceiving the meaning of life (U) |
|  |  | Improvement of capabilities (U) |
|  |  | Cognitive transformation (U) |
|  |  | Perceiving support from others (U) |
|  |  | Resilience (U) |
|  |  | Treatment period: reluctantly lowering expectations and attempting to accommodate the child (U) |
|  |  | Coping behaviors (U) |
|  |  | Impact on self-perception and well-being (U) |
|  |  | Parents’ Perceived Helpfulness (U) |
|  |  | Coping strategies of parents (U) |
|  |  | Reflection and growth (U) |
|  |  | Creating a positive family environment (U) |
|  |  | Parents’ Experience (U) |
|  |  | Reliance on a single problem-solving strategy (as a starting point for growth) (U) |
|  |  | Adaptation stage: persistent caregiving difficulties (reflexive process) (U) |
|  |  | Least Helpful Parental Responses (as reflexive learning) (U) |
| **Total synthesized findings = 5** | **Total categories = 11** | **Total findings = 77** |

U, unequivocal; C, credible; NS, not supported; NSSI, non-suicidal self-injury.

**Representative supporting illustrations and source-verification audit**

This audit trail contains all verified category-level supporting quotations. Section 3.4 of the manuscript presents one concise quotation for each of the 11 categories; additional verified quotations removed from the main text are retained here to preserve the audit trail. The complete category structure remains based on all 77 extracted findings from the 17 included studies. Only wording verified directly against an accessible full-text primary report was retained; author summaries, reconstructed wording, and unverified translations were not presented as participant quotations. Reference numbers are shown in parentheses and correspond to the manuscript.

| **Category** | **Verified participant quotation** | **Participant identifier and reference** | **Location in primary report** |
| --- | --- | --- | --- |
| Category 1: Initial shock, self-blame, and crisis-related emotional responses | *“Well I had never even heard of it… so it just… scared the daylight out of me… I don’t want other parents to be as blindsided by this as I was.”* | P2, mother; (31) | Study Two, Results: Lack of knowledge about NSSI |
| Category 1: Initial shock, self-blame, and crisis-related emotional responses | *“When I discovered my daughter’s self-injurious behavior, I did not realize that she had psychological problems and did not provide timely help. If she had been treated earlier, she would not have ended up like this…”* | P1; (9) | Primary report, p. 5, Theme 1: Guilt |
| Category 1: Initial shock, self-blame, and crisis-related emotional responses | *“We blamed him because we were scared that he would commit suicide in the future too…”* | p5; (11) | Primary report, p. 5, Theme 2: Initial response |
| Category 2: Persistent distress, fear, and emotional exhaustion | *“I was worried all day. He went out and bought a knife to cut himself. If he had accidentally cut his blood vessels, he would have died…”* | P6; (9) | Primary report, p. 5, Theme 1: Anxiety and fear |
| Category 2: Persistent distress, fear, and emotional exhaustion | *“I didn’t go to bed until yesterday. I didn’t sleep much the previous few days. I was afraid she would hurt herself.”* | p20; (11) | Primary report, p. 6, Theme 3: Increased psychological pressure |
| Category 2: Persistent distress, fear, and emotional exhaustion | *“I don’t dare to leave her alone for fear that something will happen to her. I’m under a lot of pressure (sigh).”* | Participant I; (12) | Primary report, p. 5, Theme 3.2.1: Uncertainty |
| Category 3: Affiliate stigma, concealment, and parental identity threat | *“I did not tell any of my relatives, fearing that they would not understand and would say that my child had mental health issues…”* | P3; (9) | Primary report, p. 5, Theme 1: Sense of shame |
| Category 3: Affiliate stigma, concealment, and parental identity threat | *“I would tell her to hide those scars when we go out; people look at us strangely and point…. We rarely go out anymore….”* | Participant H; (12) | Primary report, p. 5, Theme 3.2.3: Stigma |
| Category 3: Affiliate stigma, concealment, and parental identity threat | *“Rarely go to class reunions anymore, you are no more successful at work, and life is a failure when you have a child at home who often self-harms.”* | Participant O; (12) | Primary report, p. 5, Theme 3.2.3: Stigma |
| Category 4: Communication barriers and changes in caregiver-adolescent interaction | *“What didn’t help was when [my husband] and I didn’t know when to approach her to talk… we always picked the wrong time… it was a burden to talk to her…”* | P3, mother; (31) | Study Two, Results: Uncertain about interaction after NSSI |
| Category 4: Communication barriers and changes in caregiver-adolescent interaction | *“You just had to be so careful… It was like walking on eggshells with her.”* | P17, mother; (31) | Study Two, Results: Uncertain about interaction after NSSI |
| Category 4: Communication barriers and changes in caregiver-adolescent interaction | *“She was defensive, even angry, towards me. Maybe because I was too strict with her in her study. Now that she’s like this (cutting the wrists), I don’t want to ask for anything anymore.”* | P10; (33) | Primary report, p. 6, Coping behaviors: Reflection and adaptation |
| Category 5: Family relationship disruption and role strain | *“I don’t think we will ever be the same or close.”* | P12, mother; (31) | Study One, Results: Changes in the parent-adolescent relationship |
| Category 5: Family relationship disruption and role strain | *“My husband didn’t used to stay at home much, but after the child’s illness, he has changed a lot and took the initiative to take over all the housework.”* | Participant J; (12) | Primary report, p. 6, Theme 3.4.2: Intimacy |
| Category 5: Family relationship disruption and role strain | *“She has already affected the second child, who is psychologically unbalanced. She says that we are too good to her sister and that everything is about her.”* | P10; (9) | Primary report, p. 7, Theme 2: Influence on other children |
| Category 6: Caregiving burden and restriction of everyday and social life | *“I quit my job and got a psychological counselor’s license myself. Now I mainly focus on treating my child’s disease. I do not care about anything else…”* | P8; (9) | Primary report, p. 7, Theme 2: Forced abandonment of social life |
| Category 6: Caregiving burden and restriction of everyday and social life | *“In addition to hospitalization and medication costs, sometimes she wants to go out to relax and go out for fun, so the financial pressure is very great…”* | p19; (11) | Primary report, p. 6, Theme 3: Increased psychological pressure |
| Category 6: Caregiving burden and restriction of everyday and social life | *“Seeing a doctor costs money, and I have to work hard to earn money and save money, and my friends say that I have emaciated a lot, which can’t be helped.”* | Participant B; (12) | Primary report, p. 5, Theme 3.2.2: Financial burden |
| Category 7: Meaning-making, attribution, and uncertainty about NSSI | *“I don’t understand the child’s self-injury. I don’t know if it’s because my remarriage is affecting her or if it’s because she wants something…”* | Participant F; (12) | Primary report, p. 5, Theme 3.3.1: Misunderstanding |
| Category 7: Meaning-making, attribution, and uncertainty about NSSI | *“Her self-injury is, I think, a kind of resistance to mental pain… I knew it was not suicide. Otherwise the cut wouldn’t be so superficial.”* | P04; (33) | Primary report, p. 5, Perceptions: Coping strategy of negative emotions |
| Category 7: Meaning-making, attribution, and uncertainty about NSSI | *“I was shocked when her teacher told me about her self-injury at school… And confusing too… Why would she do that? I knew she was upset, but… isn’t it normal kids at her age feel upset?”* | P06; (33) | Primary report, p. 5, Perceptions: Manifestation of puberty |
| Category 8: Knowledge gaps and caregiving competence deficits | *“I had no idea that she did that in response to being in pain… I thought it was just a one time… I didn’t know that it was self-injury… I had no idea.”* | P1, mother; (31) | Study Two, Results: Lack of knowledge about NSSI |
| Category 8: Knowledge gaps and caregiving competence deficits | *“The doctor said she meets the criteria for discharge, but I still don’t dare to pick her up and bring her back, I don’t know how to take care of her…”* | Participant D; (12) | Primary report, p. 6, Theme 3.3.2: Ineffective coping |
| Category 8: Knowledge gaps and caregiving competence deficits | *“I didn’t know it was a disease. My relatives thought my daughter was in a rebellious stage…”* | P4; (9) | Primary report, p. 8, Theme 4: Cultural environment |
| Category 9: Barriers in professional, school, and community support | *“Our county hospital does not have a psychiatric department. I consulted the psychology teacher at my child’s school, but this had no effect, which is why I came to a Grade III A hospital like yours…”* | P2; (9) | Primary report, p. 7, Theme 3: Limited psychiatric resources |
| Category 9: Barriers in professional, school, and community support | *“I pretty much right away tried to get her into a counsellor but… I was completely on my own…”* | P7, mother; (31) | Study Two, Results: Negative experiences with professionals |
| Category 9: Barriers in professional, school, and community support | *“Trying to find the right person… someone who specializes in this area. There’s not much out there.”* | P14, mother; (31) | Study Two, Results: Negative experiences with professionals |
| Category 10: Needs for psychoeducation, practical guidance, and multidimensional support | *“Is there a parent’s guide to what to do when your daughter cuts? … I wish I had seen it [laughs].”* | P6, mother; (31) | Study Two, Results: Lack of knowledge/psychoeducation needs |
| Category 10: Needs for psychoeducation, practical guidance, and multidimensional support | *“I hope that the medical staff will teach us how to communicate with our children, such as parenting skills and daily communication…”* | P11; (9) | Primary report, p. 8, Theme 3: Desire for hospital support |
| Category 10: Needs for psychoeducation, practical guidance, and multidimensional support | *“I hope that when my child experiences psychological problems at home, we can directly contact the doctor to help her through psychological counseling…”* | P4; (9) | Primary report, p. 7, Theme 3: Desire for hospital support |
| Category 11: Reflective growth, coping adjustment, and reconstruction of caregiving efficacy | *“After that time, I communicated with her. I said she has grown up now, I will change myself. This family can be more democratic, I will listen to her. She can be in charge sometimes.”* | P18; (33) | Primary report, pp. 6-7, Coping behaviors: Working as a team |
| Category 11: Reflective growth, coping adjustment, and reconstruction of caregiving efficacy | *“When I speak to her now, my voice is a little lower and softer. I will deliberately pay attention to the content and my way of talking to her…”* | p2; (11) | Primary report, p. 5, Theme 3: Altered parenting and communication |
| Category 11: Reflective growth, coping adjustment, and reconstruction of caregiving efficacy | *“In the past, I always thought of earning more money for her to spend, but now I realize that my daughter doesn’t need money; she needs her mother…”* | Participant C; (12) | Primary report, p. 6, Theme 3.4.1: Reflection |
| Category 11: Reflective growth, coping adjustment, and reconstruction of caregiving efficacy | *“I specifically bought a book to learn about the characteristics of adolescent psychological development and how to interact with him…”* | P15; (9) | Primary report, p. 6, Theme 1: Learning about mental illness |
