# Supplementary material for: The lived experiences of primary caregivers supporting adolescents with non-suicidal self-injury: a qualitative systematic review and meta-aggregation
Source: Front Psychiatry. 2026 Sep 30;17:1817553. doi: 10.3389/fpsyt.2026.1817553 (PMC13662121; doi:10.3389/fpsyt.2026.1817553)
Supplement: Supplementary file 5 [file Table5.docx]

**Appendix V: ConQual Summary of Findings**

| **Synthesized finding** | **Type of research** | **Dependability** | **Credibility** | **Overall confidence rating** |
| --- | --- | --- | --- | --- |
| Synthesized Finding 1: Emotional shock, persistent distress, and stigma following NSSI discovery | Qualitative | Moderate^a^ | High | Moderate |
| Synthesized Finding 2: Disruption of family relationships, functioning, and everyday life | Qualitative | Moderate^a^ | High | Moderate |
| Synthesized Finding 3: Difficulties understanding NSSI and developing caregiving competence | Qualitative | Moderate^a^ | High | Moderate |
| Synthesized Finding 4: Barriers to support and unmet needs for coordinated, empowering services | Qualitative | Moderate^a^ | Moderate^b^ | Low |
| Synthesized Finding 5: Adaptation, reflective change, and the emergence of resilience | Qualitative | Moderate^a^ | High | Moderate |

a Dependability was downgraded one level because of methodological heterogeneity and limited reporting of researcher positioning and reflexivity.

b Credibility for Synthesized Finding 4 was downgraded one level because two contributing findings were rated Credible rather than Unequivocal.

Reviewer agreement. Final credibility and ConQual ratings were reached by consensus. Separate initial ratings were not retained in a form that permitted calculation of percentage agreement or kappa.
