# Supplementary material for: The lived experiences of primary caregivers supporting adolescents with non-suicidal self-injury: a qualitative systematic review and meta-aggregation
Source: Front Psychiatry. 2026 Sep 30;17:1817553. doi: 10.3389/fpsyt.2026.1817553 (PMC13662121; doi:10.3389/fpsyt.2026.1817553)
Supplement: Supplementary file 6 [file Table6.docx]

**Appendix VI. Reports excluded following full-text eligibility assessment**

*Retrospective reconstruction and independent eligibility verification; publication cutoff: 31 December 2025*

**Excluded reports with a verified primary reason (n=43)**

| **No.** | **Report** | **Decision** | **Report-level rationale** |
| --- | --- | --- | --- |
| 1 | McDonald G, O’Brien L, Jackson D. Guilt and shame: experiences of parents of self-harming adolescents. J Child Health Care. 2007;11(4):298–310. doi:10.1177/1367493507082759. | Ineligible phenomenon | The study used a broad self-harm construct and did not establish nonsuicidal intent for all contributing episodes; NSSI-specific caregiver findings could not be separated. |
| 2 | Byrne S, Morgan S, Fitzpatrick C, et al. Deliberate self-harm in children and adolescents: a qualitative study exploring the needs of parents and carers. Clin Child Psychol Psychiatry. 2008;13(4):493–504. doi:10.1177/1359104508096765. | Ineligible phenomenon | The sample was defined by deliberate self-harm and included suicidal behaviour; findings specific to NSSI were not reported separately. |
| 3 | Oldershaw A, Richards C, Simic M, Schmidt U. Parents’ perspectives on adolescent self-harm: qualitative study. Br J Psychiatry. 2008;193(2):140–144. doi:10.1192/bjp.bp.107.045930. | Ineligible phenomenon | Eligibility was based on self-harm without an explicit restriction to nonsuicidal intent, and no separable NSSI-only caregiver findings were available. |
| 4 | Ferrey AE, Hughes ND, Simkin S, et al. The impact of self-harm by young people on parents and families: a qualitative study. BMJ Open. 2016;6:e009631. doi:10.1136/bmjopen-2015-009631. | Ineligible phenomenon | The study used an intent-inclusive self-harm framework; findings were not stratified by suicidal intent and an NSSI-only dataset could not be isolated. |
| 5 | Ferrey AE, Hughes ND, Simkin S, et al. Changes in parenting strategies after a young person’s self-harm: a qualitative study. Child Adolesc Psychiatry Ment Health. 2016;10:20. doi:10.1186/s13034-016-0110-y. | Ineligible phenomenon | This linked report drew on the same broad self-harm sample and did not report parenting findings restricted to explicitly nonsuicidal episodes. |
| 6 | Krysinska K, Curtis S, Lamblin M, et al. Parents’ experience and psychoeducation needs when supporting a young person who self-harms. Int J Environ Res Public Health. 2020;17(10):3662. doi:10.3390/ijerph17103662. | Ineligible phenomenon | The study applied an intent-inclusive self-harm definition; neither a separable NSSI subgroup nor NSSI-specific caregiver findings were reported. |
| 7 | Townsend ML, Miller CE, Matthews EL, Grenyer BFS. Parental response style to adolescent self-harm: psychological, social and functional impacts. Int J Environ Res Public Health. 2021;18(24):13407. doi:10.3390/ijerph182413407. | Ineligible phenomenon | Self-harm was the eligibility construct and caregiver findings were not stratified by suicidal intent, preventing extraction of NSSI-only evidence. |
| 8 | Hughes ND, Locock L, Simkin S, et al. Making sense of an unknown terrain: how parents understand self-harm in young people. Qual Health Res. 2017;27(2):215–225. doi:10.1177/1049732315603032. | Ineligible phenomenon | The report used broad self-harm terminology and did not provide a separable set of caregiver findings restricted to NSSI. |
| 9 | Stewart A, Hughes ND, Simkin S, et al. Navigating an unfamiliar world: how parents of young people who self-harm experience support and treatment. Child Adolesc Ment Health. 2018;23(2):78–84. doi:10.1111/camh.12205. | Ineligible phenomenon | The linked report concerned intent-inclusive self-harm, with no separately extractable NSSI-specific caregiver findings. |
| 10 | Raphael H, Clarke G, Kumar S. Exploring parents’ responses to their child’s deliberate self-harm. Health Educ. 2006;106(1):9–20. doi:10.1108/09654280610637166. | Ineligible phenomenon | Deliberate self-harm was not restricted to behaviour without suicidal intent, and NSSI-specific findings could not be disaggregated. |
| 11 | de Miranda Trinco ME, Santos JC, Barbosa A. Experiences and needs of parents of adolescents with self-harm behaviors during hospitalization. Rev Enferm Ref. 2017;4(13):115–124. doi:10.12707/RIV17008. | Ineligible phenomenon | The report examined self-harm during hospitalization without confirming an NSSI-only sample or providing separable NSSI-specific caregiver findings. |
| 12 | Rose H, Cohen K, Kinney C. Mothers’ experiences of mental health services following their children’s self-harm. Family Science. 2011;2(3):196–202. doi:10.1080/19424620.2012.665379. | Ineligible phenomenon | The report used a broad self-harm construct and did not provide separable findings restricted to NSSI. |
| 13 | Asare-Doku W, Osafo J, Akotia CS. The experiences of attempt-survivor families and how they cope after a suicide attempt in Ghana: a qualitative study. BMC Psychiatry. 2017;17:178. doi:10.1186/s12888-017-1336-9. | Ineligible phenomenon | The index behaviour was a suicide attempt, not NSSI. |
| 14 | Asare-Doku W, Osafo J, Akotia CS. Comparing the reasons for suicide from attempt survivors and their families in Ghana. BMC Public Health. 2019;19:412. doi:10.1186/s12889-019-6743-z. | Ineligible phenomenon | The study concerned suicide attempts and reasons for suicide rather than nonsuicidal self-injury. |
| 15 | Buus N, Caspersen J, Hansen R, Stenager E, Fleischer E. Experiences of parents whose sons or daughters have (had) attempted suicide. J Adv Nurs. 2014;70(4):823–832. doi:10.1111/jan.12243. | Ineligible phenomenon | The index behaviour was attempted suicide, not NSSI. |
| 16 | Daly P. Mothers living with suicidal adolescents: a phenomenological study of their experience. J Psychosoc Nurs Ment Health Serv. 2005;43(3):22–28. | Ineligible phenomenon | Participants were mothers of suicidal adolescents; NSSI-specific caregiver findings were not available. |
| 17 | Humensky JL, Gil RM, Mazzula S, Diaz S, Lewis-Fernández R. Life is precious: views of adolescents and their mothers on methods to reduce suicidal behavior in Latinas. J Sch Nurs. 2017;33(6):426–433. doi:10.1177/1059840517722590. | Ineligible phenomenon | The study focused on suicidal behaviour and its prevention rather than NSSI-specific caregiving experiences. |
| 18 | McLaughlin C, McGowan I, Kernohan G, O’Neill S. The unmet support needs of family members caring for a suicidal person. J Ment Health. 2016;25(3):212–216. doi:10.3109/09638237.2015.1101421. | Ineligible phenomenon | The target phenomenon was suicidality; NSSI-specific caregiver data were not reported separately. |
| 19 | Boussat M, Fourcade L, Mourouvaye M, Grandclerc S, Moro MR, Lachal J. Étude qualitative de l’expérience des parents après la tentative de suicide de leur adolescent. L’Encéphale. 2022;48(4):390–396. doi:10.1016/j.encep.2021.05.005. | Ineligible phenomenon | The study examined parents’ experiences after an adolescent suicide attempt, not NSSI. |
| 20 | Lantto R, Lindkvist R, Jungert T, Westling S, Landgren K. Receiving a gift and feeling robbed: a phenomenological study on parents’ experiences of brief admissions for teenagers who self-harm at risk for suicide. Child Adolesc Psychiatry Ment Health. 2023;17. doi:10.1186/s13034-023-00675-y. | Ineligible phenomenon | The clinical pathway was explicitly organized around self-harm at risk for suicide; NSSI-specific findings were not reported separately. |
| 21 | Weissinger GM, Myrick KJ, Etingen B, et al. Parents’ experiences of an adolescent suicide crisis. Int J Ment Health Nurs. 2023;32(3):917–928. doi:10.1111/inm.13137. | Ineligible phenomenon | The phenomenon was an adolescent suicide crisis rather than NSSI. |
| 22 | Greene-Palmer FN, Wagner BM, Neely LL, Cox DW, Kochanski KM, Perera KU. How parental reactions change in response to adolescent suicide attempt. Arch Suicide Res. 2015;19(4):414–421. doi:10.1080/13811118.2015.1094367. | Ineligible phenomenon | The index event was an adolescent suicide attempt, not NSSI. |
| 23 | Ye M, Hu Y, Xue Q, Liang A, Lu Z. Understanding and change—parents’ perspectives of their self-poisoning children: a phenomenological study. Int Emerg Nurs. 2021;59:101074. doi:10.1016/j.ienj.2021.101074. | Ineligible phenomenon | Self-poisoning was the index behaviour and nonsuicidal intent was not established; NSSI-specific findings could not be isolated. |
| 24 | Ferrey AE, Hawton K, Simkin S, et al. ‘As a parent, there is no rulebook’: a new resource for parents and carers of young people who self-harm. Lancet Psychiatry. 2015;2(7):577–579. doi:10.1016/S2215-0366(15)00182-0. | Ineligible report type | This was a resource/commentary article and did not present an independently analysable primary qualitative dataset. |
| 25 | Galea B, Galea M. Experiences of parents of adolescents with deliberate self-harm behaviours. Eur Psychiatry. 2018;48(Suppl):S376–S377. | Ineligible report type | Only a conference abstract was available; the report did not provide sufficient primary qualitative findings and supporting illustrations for JBI meta-aggregation. |
| 26 | Morgan S, Rickard E, Noone M, et al. Parents of young people with self-harm or suicidal behaviour who seek help: a psychosocial profile. Child Adolesc Psychiatry Ment Health. 2013;7:13. doi:10.1186/1753-2000-7-13. | Ineligible study design | The report was quantitative and did not provide a separately reported qualitative component eligible for synthesis. |
| 27 | Baetens I, Claes L, Onghena P, et al. Non-suicidal self-injury in adolescence: a longitudinal study of the relationship between NSSI, psychological distress and perceived parenting. J Adolesc. 2014;37:817–826. doi:10.1016/j.adolescence.2014.05.010. | Ineligible study design | This was a quantitative longitudinal study without a separately reported qualitative caregiver component. |
| 28 | Tschan T, Schmid M, In-Albon T. Parenting behavior in families of female adolescents with nonsuicidal self-injury in comparison to a clinical and a nonclinical control group. Child Adolesc Psychiatry Ment Health. 2015;9:17. doi:10.1186/s13034-015-0051-x. | Ineligible study design | This was a quantitative comparative study without eligible qualitative caregiver findings. |
| 29 | Xia Q, Zhang Y, Huang X. Psychological well-being and associated factors among parents of adolescents with non-suicidal self-injury: a cross-sectional study. Front Psychiatry. 2023;14:1253321. doi:10.3389/fpsyt.2023.1253321. | Ineligible study design | This was a quantitative cross-sectional study without a separately reported qualitative component. |
| 30 | Arbuthnott AE, Lewis SP. Parents of youth who self-injure: a review of the literature and implications for mental health professionals. Child Adolesc Psychiatry Ment Health. 2015;9:35. doi:10.1186/s13034-015-0066-3. | Ineligible report type | This was a literature review rather than an original qualitative study. |
| 31 | Curtis S, Thorn P, McRoberts A, et al. Caring for young people who self-harm: a review of perspectives from families and young people. Int J Environ Res Public Health. 2018;15:950. doi:10.3390/ijerph15050950. | Ineligible report type | This was a review rather than an original qualitative study. |
| 32 | Mughal F, Troya MI, Dikomitis L, et al. The experiences and needs of supporting individuals of young people who self-harm: a systematic review and thematic synthesis. eClinicalMedicine. 2022;48:101437. doi:10.1016/j.eclinm.2022.101437. | Ineligible report type | This was a systematic review rather than an original qualitative study. |
| 33 | Simes D, Shochet I, Murray K, Sands IG. A systematic review of qualitative research of the experiences of young people and their caregivers affected by suicidality and self-harm: implications for family-based treatment. Adolesc Res Rev. 2022;7:237–254. doi:10.1007/s40894-021-00164-3. | Ineligible report type | This was a systematic review rather than an original qualitative study. |
| 34 | Martin F, Ferrey A, Hobbs L, et al. Understanding the impact of children’s and young people’s self-harm on parental well-being: a systematic literature review of qualitative and quantitative findings. Child Adolesc Ment Health. 2024;29:371–384. doi:10.1111/camh.12692. | Ineligible report type | This was a systematic review rather than an original qualitative study. |
| 35 | Juel A, Berring LL, Hybholt L, Erlangsen A, Larsen ER, Buus N. Relatives’ experiences of providing care for individuals with suicidal behaviour conceptualized as a moral career: a meta-ethnographic study. Int J Nurs Stud. 2021;113:103793. doi:10.1016/j.ijnurstu.2020.103793. | Ineligible report type | This was a meta-ethnographic review and addressed suicidal behaviour, not an original NSSI-specific qualitative study. |
| 36 | Hall S, Melia YI. ‘Just pulled myself together and realised I had to be responsible’: adolescents’ experiences of having a friend who self-harms. Child Youth Care Forum. 2021;51:885–902. doi:10.1007/s10566-021-09629-x. | Ineligible participants | Participants were adolescent friends rather than primary caregivers. |
| 37 | Yip KS, Ngan MY, Lam I. An explorative study of peer influence and response to adolescent self-cutting behavior in Hong Kong. Smith Coll Stud Soc Work. 2002;72(3):379–401. doi:10.1080/00377310209517666. | Ineligible participants | The report focused on peer influence and responses rather than primary caregivers’ experiences. |
| 38 | Berger E, Hasking P, Martin G. ‘Listen to them’: adolescents’ views on helping young people who self-injure. J Adolesc. 2013;36:935–945. | Ineligible participants | Data were provided by adolescents rather than primary caregivers. |
| 39 | Lindgren BM, Åström S, Graneheim UH. Held to ransom: parents of self-harming adults describe their lived experience of professional care and caregivers. Int J Qual Stud Health Well-being. 2010;5(3):5482. doi:10.3402/qhw.v5i3.5482. | Ineligible participants | The report concerned parents of self-harming adults; a caregiver dataset restricted to young people aged 10–24 years could not be verified. |
| 40 | Spiers S, Grandclerc S, Guénolé F, Moro MR, Lachal J. Relations parents-adolescents autour des automutilations: une étude qualitative. Neuropsychiatr Enfance Adolesc. 2020;68(1):46–54. doi:10.1016/j.neurenf.2019.09.003. | Ineligible publication language | The full report was published in French and therefore fell outside the review’s prespecified English- and Chinese-language restriction. |
| 41 | Qin Y, Wu D, Liu J, Peng J, Li C. Perspectives of parents of adolescents with repeated non-suicidal self-injury on sharing their caretaking experiences with peers: a qualitative study. Front Psychiatry. 2023;14:1237436. doi:10.3389/fpsyt.2023.1237436. | Ineligible phenomenon of interest | After independent reassessment by two reviewers, the report was excluded because its primary phenomenon was parents’ willingness, motivations, and barriers concerning peer-to-peer sharing of caretaking experiences, rather than the lived experience of directly supporting an adolescent with NSSI as defined for this synthesis. |
| 42 | Simonsson O, Engberg H, Bjureberg J, et al. Experiences of an online treatment for adolescents with nonsuicidal self-injury and their caregivers: qualitative study. JMIR Form Res. 2021;5(7):e17910. doi:10.2196/17910. | Ineligible phenomenon of interest | After independent reassessment by two reviewers, the report was excluded because it evaluated participants’ experiences and acceptability of a specific online treatment; caregiver findings addressing the review’s broader day-to-day caregiving phenomenon were not reported as a separable analytic focus. |
| 43 | Christensen SH, Heinrichsen M, Møhl B, et al. Internet-delivered emotion regulation therapy for adolescents engaging in non-suicidal self-injury and their parents: a qualitative, online focus group study. Psychol Psychother. 2025;98(2):322–341. doi:10.1111/papt.12541. | Ineligible phenomenon of interest | After independent reassessment by two reviewers, the report was excluded because its qualitative analysis focused on experiences, barriers, and facilitators of a specific internet-delivered intervention rather than primary caregivers’ lived experience of providing direct support in everyday life. |

Abbreviations: NSSI, nonsuicidal self-injury; PRISMA, Preferred Reporting Items for Systematic Reviews and Meta-Analyses.
